# White-Matter Development is Different in Bilingual and Monolingual Children: A Longitudinal DTI Study

**DOI:** 10.1371/journal.pone.0117968

**Published:** 2015-02-23

**Authors:** Seyede Ghazal Mohades, Peter Van Schuerbeek, Yves Rosseel, Piet Van De Craen, Robert Luypaert, Chris Baeken

**Affiliations:** 1 Vrije Universiteit Brussel (VUB), Brussels, Belgium; 2 Radiology, Universitair Ziekenhuis Brussel (UZBrussel), Brussels, Belgium; 3 Department of Psychiatry University Hospital, Brussels, Belgium; 4 Department of Psychiatry and Medical Psychology, Ghent University, Ghent, Belgium; 5 Department of Data Analysis, Ghent University, Ghent, Belgium; University College London, UNITED KINGDOM

## Abstract

Although numerous people grow up speaking more than one language, the impact of bilingualism on brain developing neuroanatomy is still poorly understood. This study aimed to determine whether the changes in the mean fractional-anisotropy (MFA) of language pathways are different between bilingual and monolingual children. Simultaneous-bilinguals, sequential-bilinguals and monolingual, male and female 10–13 years old children participated in this longitudinal study over a period of two years. We used diffusion tensor tractography to obtain mean fractional-anisotropy values of four language related pathways and one control bundle: 1-left-inferior-occipitofrontal fasciculus/*l*IFOF, *2*-left-arcuate fasciculus/*l*AF/*l*SLF, *3-bundle* arising from the anterior part of corpus-callosum and projecting to orbital lobe/AC-OL, *4*-fibres emerging from anterior-midbody of corpus-callosum (CC) to motor cortices/AMB-PMC, 5- right-inferior-occipitofrontal fasciculus *r*IFOF as the control pathway unrelated to language. These values and their rate of change were compared between 3 groups. FA-values did not change significantly over two years for *l*AF/*l*SLF and AC-OL. Sequential-bilinguals had the highest degree of change in the MFA value of *l*IFOF, and AMB-PMC did not present significant group differences. The comparison of MFA of lIFOF yielded a significantly higher FA-value in simultaneous bilinguals compared to monolinguals. These findings acknowledge the existing difference of the development of the semantic processing specific pathway between children with different semantic processing procedure. These also support the hypothesis that age of second language acquisition affects the maturation and myelination of some language specific white-matter pathways.

## Introduction

Since multiple language societies are expanding, with increasing numbers of families with bilingual children, the interest of the scientific community to study the effect of dual language acquisition on cerebral structure, functionality and performance in children has grown. Second language acquisition situations (explicit vs. implicit learning, the age of exposure to second language and more) have been shown to cause differences in second language proficiency by many researchers [1]. There is some evidence that speaking more than one language alters cognitive [2] and attention network processes [3]. This ability may have a beneficent effect on the maintenance of brain integrity with age [2,4–8]. The bilingual’s ability to simultaneously activate two languages, to continuously switch between them, and to inhibit the non-target language [9], can be expected to positively affect the brain structures and networks underlying language and cognition processes [10,11].

The structure-function relation of the brain has been investigated in a number of bilingualism studies. In a study of brain integrity in the elderly, it was demonstrated that the integrity of bilinguals’ brain has a slower rate of decline compared to monolinguals [6]. In another study examining cerebral atrophy in Alzheimer disease, comparing bilingual patients with monolingual controls, computed tomography (CT) showed increased atrophy in bilinguals in areas used to distinguish AD; this shows that bilingualism alters the structure of specific brain regions [12].

Nevertheless, the study of bilingualism and its impact on brain neuroanatomy remains obscure. One of the unanswered questions refers to the differences in brain microstructure development between bilinguals and monolinguals. Because brain development and maturation occurs to a large extent in childhood, a direct approach to investigating maturational changes of bilingualism in terms of the functionality of the pathways involved needs follow-up studies that compare the microstructure of the brain at different time points in children exhibiting relevant functional differences.

Brain adaptation to a changing environment, known as plasticity [13,14], is altered by bilingualism both in the gray matter volumes [11], the microstructure of white-matter pathways [15], and the cross-sectional area of sub-regions of the corpus callosum [16]. However, none of these studies investigated the developmental changes in the microstructural correlates of bilingualism; and there has been no structural brain imaging study in bilingual children. Further, the age of L2 acquisition is commonly overlooked in present studies. To the best of our knowledge, concerning the impact of bilingualism on the microstructural changes in white-matter, no such quantitative longitudinal studies have been carried out.

Diffusion tensor imaging (DTI) has been used as a non-invasive tool to characterize white-matter microstructure in vivo [17]. It provides quantitative information about the integrity and maturation of the brain through quantities like fractional anisotropy (FA), apparent diffusion coefficient (ADC) [18–20] and can be used to study the development of white-matter pathways [21]. Recently, longitudinal DTI has been used to study the effect of normal aging [22–24].

In their longitudinal DTI study in children Yeatman et al (2012) have found active biological processes in some white-matter tracts, which significantly differed according to their reading skills. Here they reported that children with higher reading proficiency had a lower MFA value on the pathways lAF and lIFOF which increased over time; while those with below-average reading skills yielded a higher initial MFA with a fast decline over time [25]. In another longitudinal study on dyslexic children, Hoeft et al (2011) predicted reading improvement based on variation in brain function and structure [26]. Treit and colleagues (2013) also reported a correlation between the changes in mean diffusivity and reading ability of patients with FASD (Fetal Alcohol Spectrum disorders) [27]. These findings reinforce the hypothesis that the functionality of specific pathways may impact the way these pathways mature.

In the current longitudinal tractography study, we have examined the development of the microstructure of four language-processing-related pathways in elementary school children using magnetic resonance diffusion tensor imaging (MR-DTI). We included three groups of subjects: simultaneous and sequential bilinguals (SimBils and SeqBils), and monolinguals (Monos). Simultaneous bilinguals were defined as individuals who acquired both their languages in parallel from birth, while sequential bilinguals were exposed to their second language from the age of three (at school). The choice of language-related pathways was motivated in our earlier study [10]. In short, the first pathway is the left inferior occipitofrontal fasciculus (*l*IFOF), which provides the ventral connection for language processing and is involved in semantic processing [28–30]. The second comprises the left arcuate fasciculus/superior longitudinal fasciculus (*l*AF/*l*SLF), the dorsal stream [31], connecting Wernicke’s area to Broca’s area [32]. The third is the bundle connecting the anterior part of the corpus callosum to the orbital lobe (AC-OL). And the fourth is the bundle of fibres emerging from the anterior midbody (AMB) of the corpus callosum that are associated with the premotor and supplementary motor cortices (AMB-PMC). This part of the corpus callosum was reported to have a larger mid-sagittal area in bilinguals as compared to monolinguals [16]. We added right inferior occipitofrontal fasciculus (*r*IFOF) as a non-linguistic pathway to act a control bundle.

In our previous study of the impact of bilingualism on the microstructure of language-specific pathways [10], it was observed that MFA value of the lIFOF and AC-OL differed significantly between the two groups of bilinguals and monolingual controls. SimBils showed the highest and Monos had the lowest MFA on the lIFOF bundle. While it was demonstrated that the bundle AC-OL has higher MFA in Monos compared to SimBils. In all cases the MFA of the bundles in SeqBils was amid the two other groups.

Our hypothesis driven aim was to supplement our earlier results with data on the diffusion anisotropy after an interval of about two years following the first observations. We expected the differences between the 3 language groups observed at the beginning of the two-year period (as documented in [10]) to be confirmed at the end of the interval. In addition, based on the findings from previous longitudinal studies [25–27,33] that report the impact of specific functions on white-matter development, we hypothesized that functional differences between the language groups would affect the maturation rate of their language related pathways. Especially the *l*IFOF, which is involved in semantic processing, was expected to reflect maturation rate differences: bilinguals have to process more semantic data (in 2 different languages) and are therefore especially dependent upon efficient neural connections in this bundle [34–36].

## Methodology

### Participants

Forty healthy male and female children who also served in our first study [10] were reassessed after an average time interval of 2 years (19–27 months, mean: 22.3 months; SD: 2.3 months). The female to male ratio was 1/1 (see Table 1). As before, the children were subdivided in 3 groups with equal variances (14 simultaneous bilinguals, 16 sequential bilinguals and 10 monolinguals). All subjects used French or Dutch as their first language (L1) and the second language (L2) of the bilinguals was restricted to Roman or Germanic languages, both branches of the Indo-European language family.

**Table 1:** Language group information.

Group	Age2 [Months] (Max-Min) Mean (SD)	Age1 [Months] (Max-Min) Mean (SD)	Gender (F/M)	Time interval [Months] (Max-Min) Mean (SD)
Simultaneous bilinguals	(118–165) 137 (11)	(99–141) 113 (11)	6/8	(19–37) 23 (5)
Sequential bilinguals	(116–154) 136 (12)	(96–131) 115 (11)	9/7	(19–24) 22 (2)
Monolinguals	(120–148) 133 (10)	(100–124) 111 (9)	5/5	(19–25) 22 (3)

The study had been approved by the Ethics Committee of our University Hospital (UZBrussel, Belgium) and informed written consent was obtained from all parents and the documents were recorded.

### DTI and MRI acquisition

The DTI and 3D MR data were acquired on a Philips Achieva 3T MR scanner with sequence parameters as previously described [10]: DTI was based on a single-shot, echo-planar Stejskal-Tanner sequence with 15 non-collinear diffusion gradients [b = 700; TR/TE = 6484ms/ 60ms; FOV = 224x224x120 mm^3^ covering 60 oblique axial with no gap and 1.75x1.75 x 2 mm^3^ resolution, total scan duration = 454s; number of averages per scan = 4]. This was supplemented by a 3D anatomical scan consisting of a T1- weighted turbo-field-echo sequence [TR/TE = 12ms/3.75ms; FOV = 200x200x200 mm^3;^ 100 axial slices; 1x1x2 mm^3^ resolution; total scan duration = 383s].

### DTI analysis

As in our previous paper [10], within slice registration was performed to correct for eddy current distortion. The DTIStudio program was used to analyze the registered images and to produce corresponding Fractional Anisotropic maps [37]. A detailed description of using the PAR/REC files and deriving FA maps was reported in [10].

**Fig 1:**
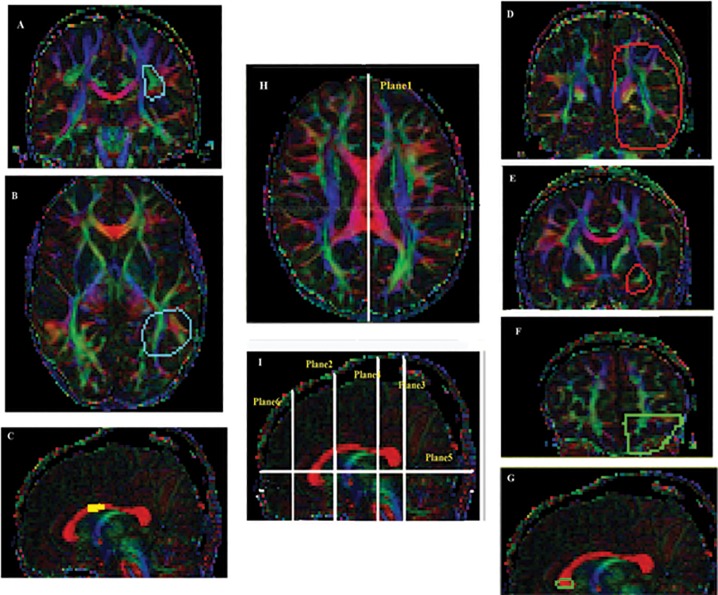
Location of ROIS to trace specific fibres; plane1: mid-sagittal slice; plane2: a coronal slice located half-way between the genu of the CC and the anterior margin of the insula; plane3: a coronal slice behind the splenium of the CC; plane4: a coronal slice at the level of the rolandic operculum; plane5: an axial slice lateral to the ventricular trigone; plane6: a coronal slice between the anterior edge of the CC and the anterior end of cerebral hemisphere Cyan ROIs (A: plane4 and B: plane5) are those to trace *l*AF/*l*SLF [81], green ROIs (F: plane6 and G: plane1) to trace AC-OL [82], the yellow ROI (C: plane1) to trace AMB-PMC [83] and the red ROIs (D: plane3 and E: plane2) to trace *l*IFOF [44] [Taken from our previous study[10]].

The FACT-algorithm (Fibre Assignment by Continuous Tracking) [38] was applied to reconstruct the 3d tracts, responsible for language processing. Each tract started from the centre of a voxel with an FA value exceeding 0.2, proceeded along the eigenvectors of neighbouring voxels and ended when the FA became smaller than 0.2 or when the tract deviated by more than 40 degrees. In order to select the desired fibre bundles, we used a region-of-interest (ROI) based approach (Fig. 1). The four selected bundles were the *l*AF/ *l*SLF, the *l*IFOF and the AC-OL, which were tracked by a two-ROI approach, and the AMB-PMC, which was found by means of a single ROI.

These ROIs had been saved for each subject in the first study (at time T1), and in order to have an accurately identical tracking in the follow-up study (at time T2), the same ROIs were used there. To this end, the 3D anatomical scans for both time points were normalized and registered to a customized paediatric template that was made based on the age and gender distribution of our study sample (at both time-points). In order to minimize the risk of errors due to using available templates that are based on adult population, Template-O-Matic toolbox was used to create the customized template using the age of subjects at both time-points and also the gender distribution [39].

The resulting normalization parameters were used for registering the diffusion images and corresponding FA maps of the both waves of scans to the template. Thus the risk of miss-localization of ROIs due to brain growth was minimized. The ROIs from T1 were then loaded on the images of T2, avoiding any manual redrawing of the ROIs.

### Quantitative and statistical analysis

The fibre tracking data were analyzed using SPSS version 20.0 (SPSS Inc, Chicago, IL USA). For each bundle and for each subject, we computed the mean FA-value (MFA) and its standard deviation. The Shapiro-Wilk test was used to assess the normality of the resulting data distribution in each language group. The majority of these distributions were not normal.

To test for gender differences, we used the two-sample t-test to compare the MFA values at time 1, the MFA values at time 2, and the change between the two MFA values (ΔMFA).

To test our main research question, a repeated measures ANOVA was applied to assess the main effect of language group ('Group'), the main effect of the time interval ('Time') and also the interaction effect ('Time x Group'). A separate analysis was done for each of the four bundles. Finally, to explore the relationship between the time interval between the two observations (ΔT = T2—T1) and the corresponding degree of change in MFA values (ΔMFA), we computed bootstrapped Pearson correlation values.

To deal with non-normality, we used bootstrapping (Efron & Tibshirani, 1994) both to assess the significance of the test statistics, and to report confidence intervals for the mean differences in follow-up post-hoc tests. All post-hoc tests were Bonferroni corrected to control for type l error and in all tests P-values smaller than 0.05 were assumed to indicate significance.

## Results

Four participants (3 SeqBils and 1 SimBil) were excluded from further analysis due to the fact that the ROIs on the FA maps for both time points could not be perfectly matched and thus the bundles could not be traced correctly.

### Gender

The two-sample t-tests did not reveal any significant differences between males and females. Only the MFA values of the lIFOF in the second time point had a marginally significant difference (F>M), t (36) = 2.02, P = 0.051.

### *l*IFOF

Here, a significant main effect of time was revealed, F (1, 35) = 49.94, P < 0.01 with a large effect size η^2^ = 0.588. We also found a significant main effect of Group, F (2, 35) = 6.51, P <0.01 and η^2^ = 0.277.

One way ANOVA revealed a significant group difference for this bundle in the second wave of scans F (2, 35) = 3.455, P = 0.043. Bonferroni corrected Post-Hoc comparisons for lIFOF-2nd showed a significant difference between Monos < SimBils (P = 0.038).

The interaction effect 'Time x Group' was also significant, F (2, 35) = 3.432, P = 0.044 with an effect size of η^2^ = 0.164. Bonferroni corrected Post-hoc comparisons revealed a significant group difference between simultaneous > sequential bilinguals (P = 0.028). The comparison between SimBils> Monos also led to a significant difference (P = 0. 006). But no significant difference could be observed in the comparison between Monos and SeqBils (P = 0.919).

The correlation analysis indicated that the ΔMFA value of lIFOF pathway was positively correlated with ΔT (correlation coefficient = 0.722, P = 0.03).

**Fig 2:**
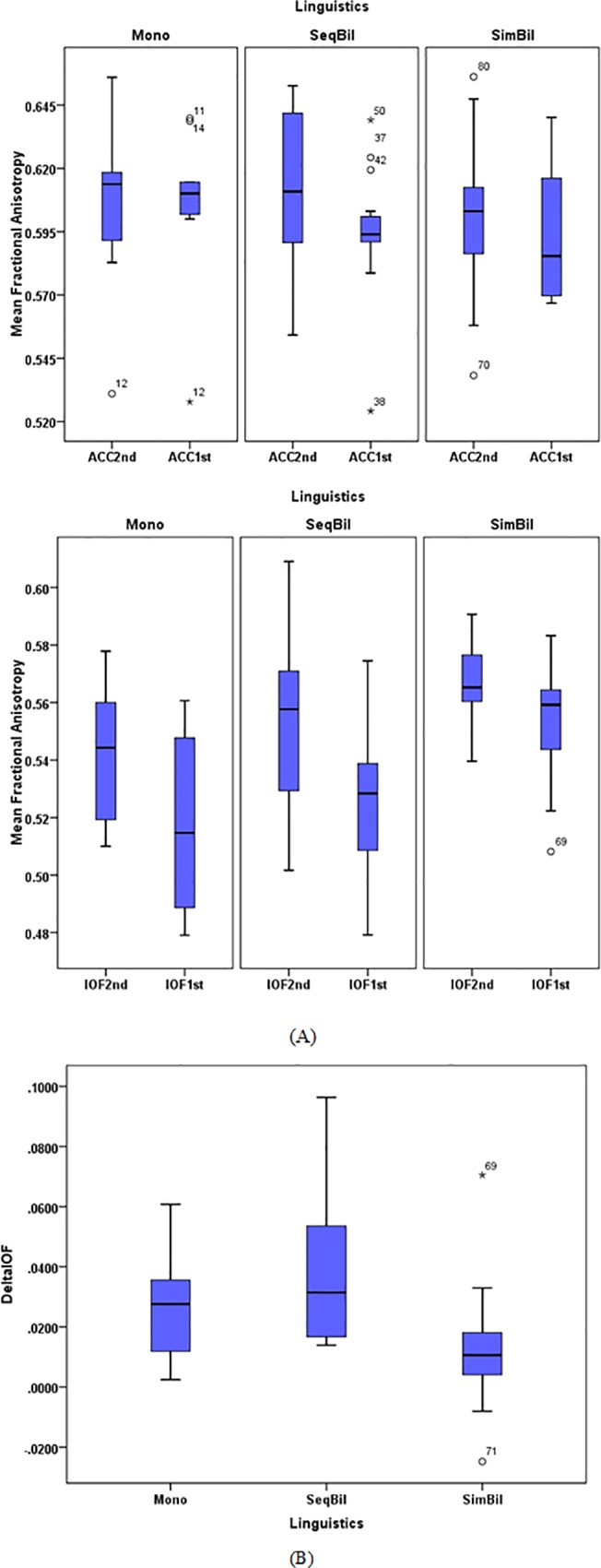
Distribution of MFA values and ΔMFA for the 3 groups A) Distribution of MFA values of the bundles AC-OL and *l*IFOF for the 3 groups and 2 time-points B) ΔMFA of the bundle *l*IFOF for the 3 groups.

ANOVA test also showed a significant group difference for ΔMFA value of the bundle lIFOF F (2, 35) = 3.432, p = 0.044. Table 2 summarizes the results of Post-hoc Bonferroni corrected tests. (Fig. 2B shows the Delta IFOF value for the bundle lIFOF in 3 groups). These show that the MFA value of the bundle lIFOF has changed the most in SeqBils compared to the 2 other groups.

**Table 2:** Post-Hoc comparison for Interaction effects using ΔMFA Values for the bundle lIFOF.

Multiple Comparisons					Bootstrap		
	(I) Group	(J) Group	Mean Difference (I-J)	Sig.	Std. Error	95% Confidence Interval	
						Lower Bound	Upper Bound
DeltaIOF	Mono	SeqBil	-0.0139	0.58	0.0098	-0.0352	0.0052
		SimBil	0.0125	0.67	0.0078	-0.0043	0.0284
	**SeqBil**	Mono	0.0139	0.58	0.0098	-0.0039	0.0328
		**SimBil**	0.0264*	**0.03**	**0.0102**	**0.0077**	**0.0445**
	**SimBil**	Mono	-0.0125	0.67	0.0078	-0.0267	0.0021
		**SeqBil**	-0.0264*	**0.03**	**0.0102**	**-0.0483**	**-0.0058**

* The mean difference is significant at the 0.05 level.

Bootstrap results are based on 1000 stratified bootstrap samples.

Fig. 2A represents the MFA values of the lIFOF for the three groups and two runs.

### lAF/lSLF

There was a significant main effect of Time for this bundle, F (1, 35) = 28.67, P < 0.01. However, there was no significant effect for Group, F (2, 35) = 0.02, *P* = 0.98, and no interaction effect ('Group x Time'), F (2, 35) = 1.19, P = 0.32. The ΔMFA values of the lAF/lSLF bundle did not correlate significantly with ΔT, correlation coefficient = 0.103, *P* = 0.54.

### AC-OL

For the AC-OL, no significant differences were found between the two time points, F (1, 35) = 2.58, P = 0.12. There was also no main effect of Group, F (2, 35) = 0.35, *P* = 0.71 and no interaction effect, F (2, 35) = 1.19, *P* = 0.32. The ΔMFA values of this bundle did not correlate significantly with ΔT, correlation coefficient = 0.06, *P* = 0.72.

### PMC

For this bundle, a significant main effect of Time was revealed, F (1, 35) = 57.3, *P* < 0.01. However, we did not find any significant group differences, F (2, 35) = 0.98, *P* = 0.39, and no interaction effect, F (2, 35) = 0.008, *P* = 0.99. The correlation analysis indicated that the ΔMFA value of the PMC pathway was not significantly correlated with ΔT, correlation coefficient = 0.01, *P* = 0.54.

### *r*IFOF

The MFA value of this control pathway did not show any significant difference between the three groups in one-way ANOVA for either of the two waves of scans. The comparison for *r*IFOF-1st run resulted in F(2,35) = 0.863, P = 0.4307 and the second run F(2,35) = 1.532, P = 0.2302.

In repeated measures ANOVA a significant effect of time was revealed, F (1, 35) = 27.1, *P* = 0.02, whereas no effect of language group F (2, 35) = 0.35, *P* = 0.67, and no interaction effect, F (2, 35) = 0.03, *P* = 0.9705 was found for this bundle

## Discussion

Based on our hypothesis that speaking more than one language affects the maturation of the brain, we conducted a 2 year longitudinal DTI study examining the mean fractional anisotropy, MFA, of four distinct language- related pathways in simultaneous and sequential bilingual and monolingual children. The first wave of scans was conducted at an age around 9 years (T1) and the second wave after a time interval of about two years (T2).

We have limited our investigations of the fibres AF/SLF and IFOF to the left hemisphere based on the left lateralization of language in more than 95% of right-handed people [40,41] and also the fact that the role of lIFOF and lAF in language processing is well established [30,42–45].

### The results of the 1st cross-sectional study

The data obtained at T1 already gave rise to an earlier cross-sectional report focused on the bilingualism-related differences in the white-matter of children between the ages of 8–10 years [10].

Using parametric tests it showed that the lIFOF and AC-OL were characterized by MFA values that were significantly different between the monolinguals and the two groups of bilinguals.

### The results of the follow-up study

With the exception of the AC-OL, all bundles showed at least marginally significant changes in MFA over the period of two years.

For the AC-OL, the group comparisons confirmed the difference in MFA between the language groups encountered at T1 in our previous study. However, this difference could no longer be established at time T2. The absence of significant group differences in the MFA of *l*AF/*l*SLF and PMC at T1 was confirmed at T2. The MFA of the lIFOF showed significant differences between the language groups at both T1 and T2. Also ΔMFA for this bundle differed significantly between the groups. Post-hoc tests showed these differences could be restricted to the comparisons SeqBil vs. SimBil in the case of ΔMFA and SimBil vs. Mono in the case of MFA2.

### lIFOF

Among widely so many areas in the temporal, parietal and frontal parts of the brain which are involved in semantic processing [29,46,47], the left inferior fronto-occipital fasciculus (IFOF) shows significant correlation with mostly all semantic procedures [48]. The lFOF is part of an important subcortical structure underlying the “semantic processing network” [29,49].

This longitudinal DTI study indicates that bilingual children, who have different semantic processing procedures compared to monolinguals, have a different rate of developing the bundle IFOF which is a semantic processing specified pathway. As at T1 we showed significant bilingualism—related differences in the MFA values for this bundle [10], The bundle *l*IFOF was of main interest of the present study as we expected it to be most affected by bilingualism. IFOF connects the prefrontal brain regions to the occipital lobes and is of major importance for language processing. The dorsolateral prefrontal cortex is a part of language-control network which is specifically activated in bilingual language tasks [50]; and the temporal and occipital lobes process the visual and audio language stimuli. Subcortical stimulation studies have demonstrated the participation of IFOF in semantic processing [29], while a picture-word-matching experiment has confirmed its role in visual-verbal incongruence judgment [51]. Its established role in word recognition and reading ability [52] and our current findings further confirms the importance of this pathway for language processing.

Recently, a clear correlation has been reported between the integrity of inferior longitudinal fasciculus (ILF) and object recognition in children [53]. This pathway is also known to carry visual information from the occipital brain regions to the temporal lobes [54] and to link the representation of objects to their lexical labels [55,56]. As there is a strong correlation between ILF and IFOF [57], this indicates that IFOF may also play a role in the process of object recognition [58]. Researchers of early language pay special attention to children under 2 year playing with objects [59–62]. They link the substitution of objects in this play (e.g. using a stick as a sward) [59], to early language development [60,61,63].

The significant group difference in the ΔMFA values of the *l*IFOF bundle suggests different maturation rates of this bundle for the three groups.

In this longitudinal study we found significant changes in MFA value of IFOF over 2-year time; reflecting the alterations in fibre characteristics during this period. The findings in the second wave of scans are consistent with the previous cross-sectional study [10] that showed significantly higher MFA values of the bundle lIFOF in bilinguals compared to monolinguals. Furthermore, Lebel and colleagues have reported that many fibres—such as SLF and IFOF—reach their maximal anisotropic changes between 13 and 20 years [64]. Compatible to their findings, in our study, the lIFOF bundle also showed significant increases in MFA value. Moreover, our results suggest that these changes are associated with linguistic skills and with age of SLA in children. In other words, maturation of this fibre seems to be related to semantic differences in bilingual children’s linguistic skills and also associated with the duration of being bilingual. Nevertheless, a direct assessment of semantic skills and correlation with MFA changes would be necessary for a more precise interpretation.

### lAF/lSLF

In our previous study [10], we had found that the bundle *l*AF/*l*SLF could not be reconstructed in all cases. This fact was explained by the incomplete myelination in the subjects involved, a result of the relatively slow rate of maturation of this specific tract (the myelination process of this bundle was reported to continue until the 3^rd^ decade of life [65,66]. Although this bundle did not show significant difference between the 3 groups in either of the two waves of observations, the fact that the MFA values of the two groups of bilinguals changed significantly over the two years, while it did not change in monolinguals supports the idea that the maturation of this bundle occurs faster in bilinguals compared to monolinguals.

### AC-OL

Our observation that the AC-OL bundle did not differ significantly between the three groups at T2, in spite of the higher MFA in monolinguals compared to bilinguals at T1, could indicate that the bilinguals have caught up with the monolinguals regarding the development of these fibres passing from the anterior part of the corpus callosum to the orbital lobe, however more specific and precise interpretation of the lack of previously seen differences is only possible by replication of this study with more participants, longer time intervals between the two scan time points. With a relatively short (~2 years) interval between T1 and T2, certain tracts may undergo late or protracted myelination in young children.

The role of this bundle in language processing is still ambiguous, however, there is a reported relationship between the degree of brain lateralization and the size of anterior corpus-callosum [67]. The established correlation between language lateralization and the size of the corpus-callosum [68,69], and also the difference in the degree of language lateralization between bilinguals and monolinguals [70], strengthens the contribution of the bundle AC-OL to bilingualism. The proven role of orbitofrontal cortex (OFC) in understanding others’ mental state [71], receiving input from audio stimuli [72], and sentence completion tasks [73] makes this part of the brain particularly important for linguistic studies. Moreover, in a condition of deliberately selecting a word that fits a sentence and obeys semantic or syntactic rules, the OFC activation is clearly observed [73]. Monitoring two languages at the same time and the need to select the suitable word between the two languages makes this selection process more crucial for bilinguals and causes difference in the maturation rate of fibres passing this area of their brain compared to monolinguals.

### AMB-PMC

In case of the bundle PMC, the tests did not result in significant difference between the MFA values of the 3 groups at the time T1 and T2. This shows that challenging our assumptions, we may have to reject the hypothesis that bigger cross-sectional anterior mid-body of the CC [16] is originated from different diffusion characteristics of fibres. However, the possible lack of statistical power may increase the risk of type 2 errors in the interpretation of the findings of this bundle [74].

Luders et al (2010) investigated the structural changes of the corpus callosum in a large group of children and adolescents aged 5–18years. They found significant correlations between callosal thickness and age suggesting that the alternating size of corpus callosum may be related to permanent change of fibres connecting homologous cortices in this range of age [75]. Other study groups, also considering wide ranges of age and a large populations, and have reported a continuous maturation of the CC throughout the lifespan [76,77].

### *r*IFOF

The right IFOF in this study was chosen as a non-language pathway to serve as a control tract. Having no evidence about the role of *r*IFOF in language circuitry one might consider the maturation of this bundle independent of linguistic differences among the three groups. The significant change in the MFA value of this pathway over the two years and the lack of observed group differences indicates that the maturation process of this bundle over the two-year time interval is not influenced by either bilingualism or age of SLA.

### Age of Second language acquisition and its impact on maturation of fibres

The observation that sequential bilinguals showed the highest degree of change in MFA among the three groups is in line with our hypothesis that the age of SLA affects the microstructural development in the brain of children. Sequential bilinguals learn their second language at the age of three or four from scratch, and pass four stages to reach full proficiency in the L2 which are: home language use, observational and listening stage (non-verbal stage), telegraphic and formulaic speech, and finally productive language use [78,79]. Completing these four stages may last from six month to two years taking into account the individual skills and the quality of L2 learning [80]. Thus at the age of 8–10, when we conducted our first wave of scans, it had been around 3–6 years that SeqBils had full proficiency in their L2 and were considered balanced bilinguals, whereas SimBils at that age had been balanced bilinguals for 8–10 years. At the time of our second wave of scans, bilinguals had 2 more years of full proficiency in their L2 and this counted up to 5–8 years for SeqBils and 10–13 years for SimBils. Compared to the first wave, the proportional difference in the length of proficiency was higher in SeqBils than in Simbils indicating stronger changes over two years in these parts of the brain in SeqBils compared to SimBils. In short, the magnitude of change in MFA of language fibres, and most evident the *l*IFOF is directly related with the years of being bilingual. Sequential bilinguals presented the highest degree of change in the MFA value of their language pathways. In SeqBils, compared to SimBils, the proportional duration of being bilingual $\left(\frac{\text{Years of being bilingual at T2}}{\text{Years of being binlingual at T1}}\right)$ was higher reflecting a higher ΔMFA value for this group.

### Conclusions, future perspectives and Limitations

The results of this study prompt the important conclusion that not only speaking more than one language, but also the age of L2 acquisition affects maturational differences and changes in the white-matter pathways involved in language processing. In spite of the relatively short time-interval between the two waves of observations, these observations support our research hypothesis indicating that language workloads has a significant impact on their myelination and aging process. Language groups that have a non-similar way of using specific pathways tend to exhibit differences in both their MFA at a given age and the rate at which these MFA change with age.

The most important limitation of this study was the time-interval of 2 years between two waves of the scans, which was chosen in such a way that, at the same time, it allowed detectable changes in the brain and fitted perfectly in our 4-year research project.

A more balanced group design, in larger populations and denser and longer time coverage would increase our understanding of bilingualism-induced developmental differences in the microstructure of white-matter throughout life.

Another point of interest in such longitudinal studies is to look into gender differences and the possible interaction of such differences with the change in the maturational characteristics of the brain. Having larger samples and more powerful statistical comparisons, this may provide a clearer insight about the influence of sex differences in brain maturation.

Another limitation of the current study was that, as the participants were chosen to be balanced proficient bilinguals in the first wave of experiments, the language tests were not re-assessed in the second run. It could be sequential bilinguals became more balanced in their knowledge or use of both languages even in relation to simultaneous bilinguals, and these changes might have been correlated with WM microstructural changes. However, as we did not include the appropriate behavioural measurements in run two this assumption should be interpreted cautiously.

Two methodological limitation regarding fibre-reconstruction in human brain are relative dependence on initial position of ROIs and also dealing with kissing, crossing and fanning fibres. Tract-editing based on prior anatomical knowledge of the specific pathways makes reconstruction results less dependent on the location of ROIs. Together with prior knowledge, terminating the tracking procedure by encountering non-cigar shaped anisotropy (we used FA>0.2) can overcome the problem of crossing and fanning fibres to a reasonable extent [38]

Of note, the pathways currently studied here are surely not the only important ones in language processing and bilingualism related issues. Further studies may do well to examine additional pathways to improve our understanding of bilingualism-induced changes on the maturation of white-matter microstructure.

In order to get a better insight about the developmental differences between the groups in more specific language regions, whole brain analysis techniques e.g. Voxel based morphometry (VBM) or TBSS could be applied.

## Supporting Information

S1 Dataset Raw data. (SAV)

S1 Highlights (DOCX)

## References

[1] Butler YG, Hakuta K (2008) Bilingualism and Second Language Acquisition The Handbook of Bilingualism: Blackwell Publishing Ltd. pp. 114.

[2] Emmorey K, Luk G, Pyers JE, Bialystok E (2008) The source of enhanced cognitive control in bilinguals: evidence from bimodal bilinguals. Psychol Sci 19: 1201–1206. 10.1111/j.1467-9280.2008.02224.x 19121123 PMC2677184

[3] Costa A, Hernandez M, Sebastian-Galles N (2008) Bilingualism aids conflict resolution: evidence from the ANT task. Cognition 106: 59–86. 17275801 10.1016/j.cognition.2006.12.013

[4] Bialystok E, Craik FI, Luk G (2012) Bilingualism: consequences for mind and brain. Trends Cogn Sci 16: 240–250. 10.1016/j.tics.2012.03.001 22464592 PMC3322418

[5] Bialystok E, Craik FIM, Grady C, Chau W, Ishii R, et al (2005) Effect of bilingualism on cognitive control in the Simon task: evidence from MEG. NeuroImage 24: 40 15588595 10.1016/j.neuroimage.2004.09.044

[6] Luk G, Bialystok E, Craik FI, Grady CL (2011) Lifelong bilingualism maintains white-matter integrity in older adults. J Neurosci 31: 16808–16813. 10.1523/JNEUROSCI.4563-11.2011 22090506 PMC3259110

[7] Takeuchi H, Sekiguchi A, Taki Y, Yokoyama S, Yomogida Y, et al (2010) Training of working memory impacts structural connectivity. J Neurosci 30: 3297–3303. 10.1523/JNEUROSCI.4611-09.2010 20203189 PMC6634113

[8] Flöel A, de Vries MH, Scholz J, Breitenstein C, Johansen-Berg H (2009) White-matter integrity in the vicinity of Broca's area predicts grammar learning success. NeuroImage 47: 1974 10.1016/j.neuroimage.2009.05.046 19477281

[9] Kroll JF, Bobb SC, Misra M, Guo T (2008) Language selection in bilingual speech: Evidence for inhibitory processes. Acta Psychologica 128: 416 10.1016/j.actpsy.2008.02.001 18358449 PMC2585366

[10] Mohades SG, Struys E, Van Schuerbeek P, Mondt K, Van De Craen P, et al (2012) DTI reveals structural differences in white-matter tracts between bilingual and monolingual children. Brain Research 1435: 72 10.1016/j.brainres.2011.12.005 22197702

[11] Mechelli A, Crinion JT, Noppeney U, O'Doherty J, Ashburner J, et al (2004) Neurolinguistics: Structural plasticity in the bilingual brain. Nature 431: 757 15483594 10.1038/431757a

[12] Schweizer T, Ware J, Fischer C, Craik F, Bialystok E (2012) Bilingualism as a contributor to cognitive reserve: evidence from brain atrophy in Alzheimer's disease. Cortex; a journal devoted to the study of the nervous system and behavior 48: 991–996. 10.1016/j.cortex.2011.04.009 21596373

[13] Pascual-Leone A, Freitas C, Oberman L, Horvath JC, Halko M, et al (2011) Characterizing brain cortical plasticity and network dynamics across the age-span in health and disease with TMS-EEG and TMS-fMRI. Brain Topogr 24: 302–315. 10.1007/s10548-011-0196-8 21842407 PMC3374641

[14] Pascual-Leone A, Amedi A, Fregni F, Merabet LB (2005) The plastic human brain cortex. Annu Rev Neurosci 28: 377–401. 16022601 10.1146/annurev.neuro.27.070203.144216

[15] Cummine J, Boliek CA (2013) Understanding white-matter integrity stability for bilinguals on language status and reading performance. Brain Struct Funct 218: 595–601. 10.1007/s00429-012-0466-6 23097036

[16] Coggins PE 3rd, Kennedy TJ, Armstrong TA (2004) Bilingual corpus-callosum variability. Brain Lang 89: 69–75. 15010238 10.1016/S0093-934X(03)00299-2

[17] Basser PJ, Mattiello J, LeBihan D (1994) MR diffusion tensor spectroscopy and imaging. Biophys J 66: 259–267. 8130344 10.1016/S0006-3495(94)80775-1 PMC1275686

[18] Madden DJ, Spaniol J, Costello MC, Bucur B, White LE, et al (2009) Cerebral white matter integrity mediates adult age differences in cognitive performance. J Cogn Neurosci 21: 289–302. 10.1162/jocn.2009.21047 18564054 PMC2676336

[19] Bava S, Thayer R, Jacobus J, Ward M, Jernigan TL, et al (2010) Longitudinal characterization of white-matter maturation during adolescence. Brain Research 1327: 38 10.1016/j.brainres.2010.02.066 20206151 PMC2854176

[20] Barnea-Goraly N, Menon V, Eckert M, Tamm L, Bammer R, et al (2005) White-Matter Development During Childhood and Adolescence: A Cross-sectional Diffusion Tensor Imaging Study. Cerebral Cortex 15: 1848–1854. 15758200 10.1093/cercor/bhi062

[21] Liu Y, Aeby A, Baleriaux D, David P, Absil J, et al (2012) White-Matter Abnormalities Are Related to Microstructural Changes in Preterm Neonates at Term-Equivalent Age: A Diffusion Tensor Imaging and Probabilistic Tractography Study. American Journal of Neuroradiology 33: 839–845. 10.3174/ajnr.A2872 22241389 PMC7968822

[22] Bennett IJ, Madden DJ, Vaidya CJ, Howard DV, Howard JH (2010) Age-related differences in multiple measures of white matter integrity: A diffusion tensor imaging study of healthy aging. Hum Brain Mapp 31: 378–390. 10.1002/hbm.20872 19662658 PMC2826569

[23] Meuter RFI, Simmond M (2007) The aging bilingual and executive function: Beyond the Simon effect. The 6th International Symposium on Bilingualism. University of Hamburg, Germany: quteprints:10401.

[24] Kennedy KM, Raz N (2009) Aging white matter and cognition: differential effects of regional variations in diffusion properties on memory, executive functions, and speed. Neuropsychologia 47: 916–927. 10.1016/j.neuropsychologia.2009.01.001 19166865 PMC2643310

[25] Yeatman JD, Dougherty RF, Ben-Shachar M, Wandell BA (2012) Development of white matter and reading skills. Proceedings of the National Academy of Sciences 109: E3045–E3053. 10.1073/pnas.1206792109 23045658 PMC3497768

[26] Hoeft F, McCandliss BD, Black JM, Gantman A, Zakerani N, et al (2011) Neural systems predicting long-term outcome in dyslexia. Proc Natl Acad Sci U S A 108: 361–366. 10.1073/pnas.1008950108 21173250 PMC3017159

[27] Treit S, Lebel C, Baugh L, Rasmussen C, Andrew G, et al (2013) Longitudinal MRI reveals altered trajectory of brain development during childhood and adolescence in fetal alcohol spectrum disorders. J Neurosci 33: 10098–10109. 10.1523/JNEUROSCI.5004-12.2013 23761905 PMC6618394

[28] Leclercq D, Duffau H, Delmaire C, Capelle L, Gatignol P, et al (2010) Comparison of diffusion tensor imaging tractography of language tracts and intraoperative subcortical stimulations. J Neurosurg 112: 503–511. 10.3171/2009.8.JNS09558 19747052

[29] Duffau H, Gatignol P, Mandonnet E, Peruzzi P, Tzourio-Mazoyer N, et al (2005) New insights into the anatomo-functional connectivity of the semantic system: a study using cortico-subcortical electrostimulations. Brain 128: 797–810. 15705610 10.1093/brain/awh423

[30] Mandonnet E, Nouet Al, Gatignol P, Capelle L, Duffau H (2007) Does the left inferior longitudinal fasciculus play a role in language? A brain stimulation study. Brain 130: 623–629. 17264096 10.1093/brain/awl361

[31] Acton QA (2012) Advances in Cerebrum Research and Application: 2012 Edition: ScholarlyEditions 10.1007/s12070-012-0514-9

[32] Brauer J, Anwander A, Friederici AD (2011) Neuroanatomical Prerequisites for Language Functions in the Maturing Brain. Cerebral Cortex 21: 459–466. 10.1093/cercor/bhq108 20566580

[33] Darki F, Klingberg T (2014) The Role of Fronto-Parietal and Fronto-Striatal Networks in the Development of Working Memory: A Longitudinal Study. Cereb Cortex. 10.1093/cercor/bht352 24414278

[34] Turken AU, Whitfield-Gabrieli S, Bammer R, Baldo JV, Dronkers NF, et al (2008) Cognitive processing speed and the structure of white matter pathways: Convergent evidence from normal variation and lesion studies. NeuroImage 42: 1032 10.1016/j.neuroimage.2008.03.057 18602840 PMC2630965

[35] Penke L, Munoz Maniega S, Murray C, Gow AJ, Hernandez MC, et al (2010) A general factor of brain white matter integrity predicts information processing speed in healthy older people. J Neurosci 30: 7569–7574. 10.1523/JNEUROSCI.1553-10.2010 20519531 PMC6632368

[36] Segura B, Jurado M, Freixenet N, Bargallo N, Junque C, et al (2010) White matter fractional anisotropy is related to processing speed in metabolic syndrome patients: a case-control study. BMC Neurology 10: 64 10.1186/1471-2377-10-64 20663196 PMC2920865

[37] Jiang H, van Zijl PCM, Kim J, Pearlson GD, Mori S (2006) DtiStudio: Resource program for diffusion tensor computation and fiber bundle tracking. Computer Methods and Programs in Biomedicine 81: 106 16413083 10.1016/j.cmpb.2005.08.004

[38] Mori S, van Zijl PCM (2002) Fiber tracking: principles and strategies—a technical review. NMR in Biomedicine 15: 468 12489096 10.1002/nbm.781

[39] Wilke M, Holland SK, Altaye M, Gaser C (2008) Template-O-Matic: a toolbox for creating customized pediatric templates. Neuroimage 41: 903–913. 10.1016/j.neuroimage.2008.02.056 18424084

[40] Bethmann A, Tempelmann C, De Bleser R, Scheich H, Brechmann A (2007) Determining language laterality by fMRI and dichotic listening. Brain Res 1133: 145–157. 17182011 10.1016/j.brainres.2006.11.057

[41] Vikingstad EM, George KP, Johnson AF, Cao Y (2000) Cortical language lateralization in right handed normal subjects using functional magnetic resonance imaging. Journal of the Neurological Sciences 175: 17 10785252 10.1016/s0022-510x(00)00269-0

[42] Balsamo LM, Xu B, Grandin CB, Petrella JR, Braniecki SH, et al (2002) A functional magnetic resonance imaging study of left hemisphere language dominance in children. Arch Neurol 59: 1168–1174. 12117366 10.1001/archneur.59.7.1168

[43] Glasser MF, Rilling JK (2008) DTI tractography of the human brain's language pathways. Cereb Cortex 18: 2471–2482. 10.1093/cercor/bhn011 18281301

[44] Rodrigo S, Naggara O, Oppenheim C, Golestani N, Poupon C, et al (2007) Human Subinsular Asymmetry Studied by Diffusion Tensor Imaging and Fiber Tracking. AJNR Am J Neuroradiol 28: 1526–1531. 17846205 10.3174/ajnr.A0584 PMC8134399

[45] Vernooij MW, Smits M, Wielopolski PA, Houston GC, Krestin GP, et al (2007) Fiber density asymmetry of the arcuate fasciculus in relation to functional hemispheric language lateralization in both right- and left-handed healthy subjects: A combined fMRI and DTI study. NeuroImage 35: 1064 17320414 10.1016/j.neuroimage.2006.12.041

[46] Cohen L, Dehaene S (2004) Specialization within the ventral stream: the case for the visual word form area. Neuroimage 22: 466–476. 15110040 10.1016/j.neuroimage.2003.12.049

[47] Price CJ (2000) The anatomy of language: contributions from functional neuroimaging. J Anat 197 Pt 3: 335–359. 11117622 10.1046/j.1469-7580.2000.19730335.x PMC1468137

[48] Han Z, Ma Y, Gong G, He Y, Caramazza A, et al. (2013) White-matter structural connectivity underlying semantic processing: Evidence from brain damaged patients. Brain. 10.1093/brain/awt205 23975453

[49] Martino J, Brogna C, Robles SG, Vergani F, Duffau H (2010) Anatomic dissection of the inferior fronto-occipital fasciculus revisited in the lights of brain stimulation data. Cortex 46: 691 10.1016/j.cortex.2009.07.015 19775684

[50] Abutalebi J, Green D (2007) Bilingual language production: The neurocognition of language representation and control. Journal of Neurolinguistics 20: 242.

[51] Plaza M, Gatignol P, Cohen H, Berger B, Duffau H (2008) A Discrete Area within the Left Dorsolateral Prefrontal Cortex Involved in Visualâ€“Verbal Incongruence Judgment. Cerebral Cortex 18: 1253–1259. 17921457 10.1093/cercor/bhm169

[52] Cohen L, Martinaud O, Lemer C, Lehéricy S, Samson Y, et al (2003) Visual Word Recognition in the Left and Right Hemispheres: Anatomical and Functional Correlates of Peripheral Alexias. Cerebral Cortex 13: 1313–1333. 14615297 10.1093/cercor/bhg079

[53] Ortibus E, Verhoeven J, Sunaert S, Casteels I, de Cock P, et al (2012) Integrity of the inferior longitudinal fasciculus and impaired object recognition in children: a diffusion tensor imaging study. Dev Med Child Neurol 54: 38–43. 10.1111/j.1469-8749.2011.04147.x 22171928

[54] Catani M, Jones DK, Donato R, Ffytche DH (2003) Occipito-temporal connections in the human brain. Brain 126: 2093–2107. 12821517 10.1093/brain/awg203

[55] Mummery CJ, Patterson K, Wise RJ, Vandenberghe R, Price CJ, et al (1999) Disrupted temporal lobe connections in semantic dementia. Brain 122 (Pt 1): 61–73. 10050895 10.1093/brain/122.1.61

[56] Catani M, Mesulam M (2008) The arcuate fasciculus and the disconnection theme in language and aphasia: history and current state. Cortex 44: 953–961. 10.1016/j.cortex.2008.04.002 18614162 PMC2740371

[57] Wahl M, Li Y-O, Ng J, LaHue SC, Cooper SR, et al (2010) Microstructural correlations of white matter tracts in the human brain. NeuroImage 51: 531–541. 10.1016/j.neuroimage.2010.02.072 20206699 PMC2856800

[58] Ashtari M (2011) Anatomy and functional role of the inferior longitudinal fasciculus: a search that has just begun. Developmental Medicine & Child Neurology 54: 6 10.1038/ni.3099 22098073

[59] Bretherton I (1984) Symbolic Play: The Development of Social Understanding: Academic Press

[60] Rescorla L, Goossens M (1992) Symbolic Play Development in Toddlers With Expressive Specific Language Impairment (SLI-E). J Speech Hear Res 35: 1290–1302. 1283611 10.1044/jshr.3506.1290

[61] Bergen D (2002) The role of pretend play in children's cognitive development: ERIC Clearinghouse.

[62] Rutherford MD, Young GS, Hepburn S, Rogers SJ (2007) A longitudinal study of pretend play in autism. J Autism Dev Disord 37: 1024–1039. 17146707 10.1007/s10803-006-0240-9

[63] Smith LB, Jones SS (2011) Symbolic play connects to language through visual object recognition. Developmental Science 14: 1142–1149. 10.1111/j.1467-7687.2011.01065.x 21884329 PMC3482824

[64] Lebel C, Walker L, Leemans A, Phillips L, Beaulieu C (2008) Microstructural maturation of the human brain from childhood to adulthood. Neuroimage 40: 1044–1055. 10.1016/j.neuroimage.2007.12.053 18295509

[65] Rauschecker AM, Deutsch GK, Ben-Shachar M, Schwartzman A, Perry LM, et al (2009) Reading impairment in a patient with missing arcuate fasciculus. Neuropsychologia 47: 180–194. 10.1016/j.neuropsychologia.2008.08.011 18775735 PMC2671152

[66] Friederici AD (2009) Pathways to language: fiber tracts in the human brain. Trends in Cognitive Sciences 13: 175 10.1016/j.tics.2009.01.001 19223226

[67] Putnam MC, Wig GS, Grafton ST, Kelley WM, Gazzaniga MS (2008) Structural organization of the corpus callosum predicts the extent and impact of cortical activity in the nondominant hemisphere. J Neurosci 28: 2912–2918. 10.1523/JNEUROSCI.2295-07.2008 18337422 PMC6670679

[68] Josse G, Seghier ML, Kherif F, Price CJ (2008) Explaining function with anatomy: language lateralization and corpus-callosum size. J Neurosci 28: 14132–14139. 10.1523/JNEUROSCI.4383-08.2008 19109495 PMC2689513

[69] Witelson SF (1989) Hand and sex differences in the isthmus and genu of the human corpus-callosum. A postmortem morphological study. Brain 112 (Pt 3): 799–835. 2731030 10.1093/brain/112.3.799

[70] Hull R, Vaid J (2006) Laterality and language experience. Laterality: Asymmetries of Body, Brain and Cognition 11: 436. 10.1080/13576500600691162 16882556

[71] Hynes CA, Baird AA, Grafton ST (2006) Differential role of the orbital frontal lobe in emotional versus cognitive perspective-taking. Neuropsychologia 44: 374 16112148 10.1016/j.neuropsychologia.2005.06.011

[72] Kringelbach ML, Rolls ET (2004) The functional neuroanatomy of the human orbitofrontal cortex: evidence from neuroimaging and neuropsychology. Progress in Neurobiology 72: 341 15157726 10.1016/j.pneurobio.2004.03.006

[73] Elliott R, Dolan RJ, Frith CD (2000) Dissociable Functions in the Medial and Lateral Orbitofrontal Cortex: Evidence from Human Neuroimaging Studies. Cerebral Cortex 10: 308–317. 10731225 10.1093/cercor/10.3.308

[74] Christley RM (2010) Power and Error: Increased Risk of False Positive Results in Underpowered Studies. The Open Epidemiology Journal 3: 16–19.

[75] Luders E, Thompson PM, Toga AW (2010) The development of the corpus callosum in the healthy human brain. J Neurosci 30: 10985–10990. 10.1523/JNEUROSCI.5122-09.2010 20720105 PMC3197828

[76] Kim EY, Kim DH, Yoo E, Park HJ, Golay X, et al (2007) Visualization of maturation of the corpus callosum during childhood and adolescence using T2 relaxometry. Int J Dev Neurosci 25: 409–414. 17964752 10.1016/j.ijdevneu.2007.05.005

[77] Paus T, Collins DL, Evans AC, Leonard G, Pike B, et al (2001) Maturation of white matter in the human brain: a review of magnetic resonance studies. Brain Research Bulletin 54: 255–266. 11287130 10.1016/s0361-9230(00)00434-2

[78] Toppelberg CO, Tabors P, Coggins A, Lum K, Burger C (2005) Differential diagnosis of selective mutism in bilingual children. J Am Acad Child Adolesc Psychiatry 44: 592–595. 15908842 10.1097/01.chi.0000157549.87078.f8 PMC3538870

[79] Tabors PO (1997) One child, two languages: a guide for preschool educators of children learning English as a second language: Paul H. Brookes Pub

[80] California Department of Education CDDicwTCfCaFS, WestEd (2012) Dual Language Learners (DLL).

[81] Rodrigo S, Oppenheim C, Chassoux F, Hodel J, De Vanssay A, et al (2008) Language lateralization in temporal lobe epilepsy using functional MRI and probabilistic tractography. Epilepsia 49: 1367 10.1111/j.1528-1167.2008.01607.x 18410362

[82] Huang H, Zhang J, Jiang H, Wakana S, Poetscher L, et al (2005) DTI tractography based parcellation of white matter: Application to the mid-sagittal morphology of corpus callosum. NeuroImage 26: 195 15862219 10.1016/j.neuroimage.2005.01.019

[83] Coggins PE 3rd, Kennedy TJ, Armstrong TA (2004) Bilingual corpus callosum variability. Brain Lang 89: 69–75. 15010238 10.1016/S0093-934X(03)00299-2

