# Supplementary material for: White-Matter Development is Different in Bilingual and Monolingual Children: A Longitudinal DTI Study
Source: PLoS One. 2015 Feb 23;10(2):e0117968. doi: 10.1371/journal.pone.0117968 (PMC4338107; doi:10.1371/journal.pone.0117968)
Supplement: S1 Highlights — (DOCX) [file pone.0117968.s002.docx]

**Highlights**

- Bilingualism affects the maturation of white matter structure
- Age of 2^nd^ language acquisition alters the maturation process of language fibres
- Delta-FA of lIFOF is different in children with different semantic skills
- Bilinguals have a higher FA value and maturation rate of specific WM pathways
